# Fungi Originating From Tree Leaves Contribute to Fungal Diversity of Litter in Streams

**DOI:** 10.3389/fmicb.2019.00651

**Published:** 2019-04-02

**Authors:** Pirjo Koivusaari, Mysore V. Tejesvi, Mikko Tolkkinen, Annamari Markkola, Heikki Mykrä, Anna Maria Pirttilä

**Affiliations:** ^1^Ecology and Genetics, University of Oulu, Oulu, Finland; ^2^Chain Antimicrobials Oy, Oulu, Finland; ^3^Pöyry Finland Oy, Oulu, Finland; ^4^Freshwater Centre, Finnish Environment Institute, Oulu, Finland

**Keywords:** endophytes, epiphytes, next-generation sequencing, aquatic fungi, litter, diversity

## Abstract

Biomass production and decomposition are key processes in ecology, where plants are primarily responsible for production and microbes act in decomposition. Trees harbor foliar microfungi living on and inside leaf tissues, epiphytes, and endophytes, respectively. Early researchers hypothesized that all fungal endophytes are parasites or latent saprophytes, which slowly colonize the leaf tissues for decomposition. While this has been proven for some strains in the terrestrial environment, it is not known whether foliar microfungi from terrestrial origin can survive or perform decomposition in the aquatic environment. On the other hand, aquatic hyphomycetes, fungi which decompose organic material in stream environments, have been suggested to have a plant-associated life phase. Our aim was to study how much the fungal communities of leaves and litter submerged in streams overlap. Ergosterol content on litter, which is an estimator of fungal biomass, was 5–14 times higher in submerged litter than in senescent leaves, indicating active fungal colonization. Leaves generally harbored a different microbiome prior to than after submergence in streams. The Chao1 richness was significantly higher (93.7 vs. 60.7, *p* = 0.004) and there were more observed operational taxonomic units (OTUs) (78.3 vs. 47.4, *p* = 0.004) in senescent leaves than in stream-immersed litter. There were more Leotiomycetes (9%, *p* = 0.014) in the litter. We identified a group of 35 fungi (65%) with both plant- and water-associated lifestyles. Of these, eight taxa had no previous references to water, such as lichenicolous fungi. Six OTUs were classified within Glomeromycota, known as obligate root symbionts with no previous records from leaves. Five members of Basidiomycota, which are rare in aquatic environments, were identified in the stream-immersed litter only. Overall, our study demonstrates that foliar microfungi contribute to fungal diversity in submerged litter.

## Introduction

Biomass production and decomposition are key processes in ecology. Whereas plants are primarily responsible for production of biomass, microorganisms together with animals decompose the organic material in various ecosystems (Gessner et al., 2010). Trees harbor foliar microfungi; epiphytes that colonize on leaf surfaces, and endophytes that live inside leaf tissues (Petrini, 1986; Lindow and Brandl, 2003; Hardoim et al., 2015), but their ecological roles, ranging from pathogenicity to mutualism, have been unknown or under dispute. Early papers hypothesized that all fungal endophytes are saprophytes or parasites, which slowly colonize the living leaf tissues and gain a head start over soil-dwelling saprophytes that invade the fallen, dead leaves (Sherwood-Pike et al., 1986; Saikkonen et al., 1998; Müller et al., 2001; Koide et al., 2005). This hypothesis was based on results made by early classification methods, leaning on morphological characteristics that erroneously classified fungal species into pathogens, saprophytes or parasites of the host tree (Berbee, 2001; Baayen et al., 2002). However, modern classification methods, which are based on sequencing of DNA and ribosomal RNA genes, have revealed that most endophytes are different from parasites of the host tree (Ganley et al., 2004).

Sequence-based analyses have shown that a specific group of endophytic strains can act as saprotrophs in the laboratory (Promputtha et al., 2007; Sun et al., 2011) or in a terrestrial environment (Unterseher et al., 2013). In general, leaf decomposition is a process taking several months where succession of different groups of decomposers occurs, endophytes being considered within the group of primary decomposers (Šnajdr et al., 2010; Vořšková and Baldrian, 2013). However, fungal endophytes can have a significant effect on leaf decomposition, whether participating in it or not. For example, the grass endophyte, *Neotyphodium* sp., inhibits decomposition of leaves of *Lolium multiflorum*, *Bromus unioloides* (Omacini et al., 2004), and *L. arundinaceum* (Lemons et al., 2005). Fungal endophytes can change host metabolism, rendering fallen leaves altered by chemical composition (Schmidt et al., 1982; Lyons et al., 1990). Altered leaf chemistry can affect decomposition rates, other microbial decomposers, invertebrate detritivores, and the overall decomposition microenvironment (Omacini et al., 2004; Lemons et al., 2005; Osono and Hirose, 2009). Fungal endophytes can also delay or prevent the colonization of host leaves by saprotrophs (Purahong and Hyde, 2011). Such effects have been observed in terrestrial (Cornelissen et al., 2000) and aquatic environments, where endophytic and pathogenic *Rhytisma* spp. inhibited decomposition of maple litter (LeRoy et al., 2011; Grimmett et al., 2012).

Diversity of foliar microfungi is intimately associated with forest management; nursery-grown trees host lower richness of endophytes compared to those growing in an old-growth forest (Ganley and Newcombe, 2006). Leaves originating from the terrestrial ecosystem and landing into an aquatic one create cross-ecosystem flows of nutrients, and the global species loss is feared to impair decomposition processes, especially in the aquatic environment (Santschi et al., 2018). Therefore, understanding factors that affect microbial diversity in aquatic litter is crucial. Aquatic hyphomycetes, fungi which decompose organic material in stream environments, have been suggested to have a terrestrial plant-associated life phase (Selosse et al., 2008; Chauvet et al., 2016). On the other hand, it is not totally clear whether foliar microfungi from terrestrial origin can thrive in the aquatic environment.

Overall, roughly 3000 fungal species have been reported from aquatic habitats (Shearer et al., 2007). Most taxa of aquatic fungi comprise the Ascomycetes, Chytridiomycetes, and mitosporic (asexual) taxa. Basidiomycetes are largely absent in aquatic environments (Shearer et al., 2007), although Basidiomycetes yeasts have been identified in submerged litter (Sampaio et al., 2007). Aquatic hyphomycetes (Ingoldian fungi) live mainly in low-order streams, and are found as saprophytes on dead plant material, such as leaves and wood (Ingold, 1942; Suberkropp, 1991; Bärlocher, 1992; Gessner et al., 1993). They are considered an ecological and not a taxonomic group (Bärlocher, 1992), but the majority of aquatic hyphomycetes are mitosporic Ascomycetes (Bärlocher, 2016) that have gone through a secondary adaptation to aquatic life (Belliveau and Bärlocher, 2005). Aquatic hyphomycetes may include resident fungi that spend full life cycles in the aquatic environment, never entering other (terrestrial) habitats, or transient fungi that are present in the water only for limited time periods (Shearer et al., 2007).

Finding of aquatic hyphomycetes present in distilled water after leaf submergence (Bandoni, 1972) and identification of aquatic hyphomycetes sequences among spruce ectomycorrhiza (Murat et al., 2005), orchid mycorrhiza (Abadie et al., 2006; Stark et al., 2009; Elena et al., 2010), orchid roots (Tedersoo et al., 2007), and liverworts (Russell and Bulman, 2005), have suggested that aquatic hyphomycetes have a plant-associated life-phase. Fabre (1998) reported the occurrence of aquatic hyphomycetes to rise in rivers after leaf fall. From black spruce, an endophyte was isolated that could also survive in the stream water environment (Sokolski et al., 2006). Similarly, Seena and Monroy (2015) made a phylogenetic analysis on fungal strains deposited to GenBank and concluded that endophytes can be aquatic hyphomycetes. But how large is the proportion of foliar microfungi that thrives in decomposing leaves in an aquatic environment, and do aquatic hyphomycetes of litter originate from the plant? Our aim was to study these questions by sequencing the fungal communities of alder (*Alnus incana* L.) leaves prior to and after submergence in pristine boreal streams. Because low water pH has previously been reported to negatively affect microbial decomposition (Mulholland et al., 1987) and fungal biomass (Ely et al., 2010), we measured ergosterol content, indicating fungal biomass, to compare fungal colonization of litter submerged in circumneutral and acidic streams.

## Materials and Methods

### Study Sites

The 12 study streams are located in the Iijoki and Oulujoki drainage basins, consisting of pristine circumneutral and naturally acidic streams. The two river basins used in this study differ by bedrock geology. The six streams located in the Oulujoki basin drain through black-shale dominated geology, which causes naturally low pH (mean pH 5.3, range 4.2–6.3) and high metal concentrations in the stream water (Table 1 and Supplementary Table S1; Loukola-Ruskeeniemi et al., 1998). The six circumneutral streams (mean pH 6.5, range 5.9–6.8) in Iijoki basin (Table 1 and Supplementary Table S1) drain through peatlands typical for Northern Finland. Streams are first-to-second order streams (base flow <0.6 m^3^ s^-1^) with similar instream habitat characteristics among the two basins (Table 1 and Supplementary Table S1). The stream sites had no obvious signs of human impact in the stream channel (e.g., channelization) or in the riparian zone (e.g., forest ditches, clear-cuts, roads within 100 m from the stream edge). The field protocols for measuring water chemistry were performed as described by Tolkkinen et al. (2013, 2015).

**Table 1:** Means and ranges of environmental variables of the study streams in the two river basins.

	Iijoki basin		Oulujoki basin	
	Mean	Range	Mean	Range
**Water chemistry**				
tot-P (μg/L)	11	7–15	20	12–24
pH	6.5	5.9–6.8	5.3	4.2–5.6
Fe (μg/L)	695	170–1000	1657	840–2200
Aluminum (μg/L)	93	48–149	295	229–331
Copper (μg/L)	0.13	0.1–0.17	0.78	0.38–1.00
Zinc (μg/L)	2.34	1.56–3.07	14.20	7.69–18.60
DOC (mg/L)	14	5–20	18	10–40
Alkalinity (mmol/L)	0.15	0.08–0.2	0.015	-0.02 to 0.05
Conductivity (mS/m)	2.7	1.9–3.6	3.3	2.6–4.6
**In-stream characteristics**				
Current velocity (m/s)	0.18	0.12–0.23	0.37	0.19–0.47
Stream depth (cm)	25	23–41	25	19–31
Stream width (m)	2.3	0.5–4.0	4.0	0.7–5.0
Moss cover (%)	75	65–87	54	18–78
Particle size^∗^	6	5–8	7	5–8

*River-specific variables are listed in detail in Supplementary Table S1. ^∗^Substrate size was based on ten estimates of the % cover of ten substrate classes: (0) organic matter, (1) sand (diameter 0.25–2 mm), (2) fine gravel (2–6 mm), (3) coarse gravel (6–16 mm), (4) small pebble (16–32 mm), (5) large pebble (32–64 mm), (6) small cobble (64–128 mm), (7) large cobble (128–256 mm), (8) boulder (256–400 mm), (9) large boulder and bed rock (>400 mm).*

### Field Procedures

All alder leaves used in the experiments were collected from the same location (sampling area 30 m × 30 m) in Oulu, Northern Finland (65°N; 25°30′E). The leaves were collected just prior to abscission in September 2010 and air-dried at room temperature for 2 weeks. We wanted to mimic the natural process of leaf senescence, abscission, and falling into the stream (Allan and Castillo, 2007) using the homogeneous material of nearly senescent leaves. Although, they can have slightly different chemistry and fungal community composition compared to naturally fallen senescent leaves (Schmidt et al., 1982; Lyons et al., 1990). Six grams of the dried leaves were enclosed in 15 cm × 15 cm mesh bags (mesh size 0.2 mm). A mesh size of 0.2 mm was used to exclude shredding invertebrates. At each experiment site, five mesh bags were anchored to the stream bed with house bricks. After 30 days, the bags were removed from each stream, sealed in zip-lock bags, and transferred to -20°C. In the laboratory, the litter bags were gently cleaned to remove other material. Subsamples of 12.5 cm^2^ were taken for extraction of DNA and for measuring ergosterol content (see description below).

### DNA Isolation, Library Construction, and Sequencing

Subsamples (five pooled 12.5 cm^2^ litter samples from each stream) from the 12 streams and three samples of originally collected senescent leaf material were analyzed. Two senescent leaf samples were studied without exposure to water and one sample was studied after immersion in distilled water to include in the analysis of dormant fungi that are activated in the presence of water (as observed by Bandoni, 1972). The fungal OTUs (operational taxonomic units) were determined using 454 pyrosequencing. DNA was extracted from 0.07 g of frozen leaf material using PowerSoil DNA Isolation Kit (MOBIO Laboratories, Carlsbad, CA, United States). The internal transcribed region (ITS) 1 of the ribosomal RNA genes of fungi was amplified using the forward primer ITS1F 5′-CTTGGTCATTTAGAGGAAGTAA-3′ (Gardes and Bruns, 1993) with the 454 pyrosequencing adaptor (Roche, Basel, Switzerland) and barcode sequences, and the reverse primer ITS4 5′-TCCTCCGCTTATTGATATGC-3′ (White et al., 1990). The amplicons were sequenced using the GS FLX 454 system (Roche, Basel, Switzerland), according to the manufacturer’s instructions at the Viikki Biocenter Sequencing Facility, University of Helsinki, Finland.

### Analysis of Fungal Communities

The sequences and quality scores were extracted from the original SFF files, and the sequencing tags were analyzed and removed using the QIIME software package (Caporaso et al., 2010). Sequence reads, 80,117 in total, were quality filtered in QIIME. Singletons, sequences shorter or longer than 200–600 bp in length, and sequences with a quality score under 25, corresponding to 3.8% of reads in total, were removed. The sequence length was trimmed to 262 bases from the forward primer to exclude the ITS2 region. UCLUST (Edgar, 2010) was used for the *de novo* OTU, picking with a sequence similarity value of 97% and for taxonomic assignments against the UNITE reference database in QIIME. Chimeric sequences, corresponding to 0.7% of reads in the dataset, were removed with “usearch” quality filter in the UNITE reference database. Completely unassigned sequences, corresponding to 52.7% of all reads in the dataset, were removed from the OTU table. All samples were rarefied to 312 sequences prior to the OTU-based diversity analysis, as it was the number of the lowest observed reads in the community. The rarefaction, relative abundance, and alpha diversity index (Chao1, Observed OTUs) analyses were done with QIIME using the rarefied OTU table. A detailed taxonomic composition of fungi from phylum to species level was visualized with Krona (Ondov et al., 2011). For the Venn diagram (drawn with BxToolBox at https://bioinforx.com/lims2/product_bxtoolbox.php), we counted the numbers of OTUs occurring consistently only in senescent leaves, stream-immersed litter, or both environments. The OTUs in different groups (leaves, litter, or both) were annotated using BLAST (The Basic Local Alignment Search Tool) searches of NCBI (The National Center for Biotechnology Information, United States) GenBank’s non-redundant nucleotide database. The next-generation sequence data has been deposited into SRA under the accession number SRP125716.

### Ergosterol Analysis

The pooled subsamples of 12.5 cm^2^ were used for measurement of ergosterol content. The weight of each subsample was recorded for calculating ergosterol content. Fungal biomass was estimated from freeze-dried and pulverized material using a modified ergosterol assay, originally described by Nylund and Wallander (1992). Ergosterol extracts were analyzed with high-pressure liquid chromatography (HPLC), using a reverse-phase C18 column equipped with a pre-cartridge. Methanol was used as the eluent (1.0 mL min^-1^) with a column temperature of 30 °C. The commercial ergosterol (5,7,22-Ergostatrien-3β-ol, Fluka AG) was used as the standard, and the results were calculated as ergosterol concentration in the litter (μg g^-1^ litter DW).

### Statistical Analyses

Non-metric Multidimensional Scaling (NMDS) based on Bray-Curtis dissimilarities of log-transformed rarefied abundance data was used to summarize the variability of fungal assemblage structure between senescent leaves and submerged litter. A two-dimensional NMDS solution was used, because reduction in stress value was minor with further dimension. The structures of fungal communities in the water-immersed litter of streams with low or neutral water pH were similar, therefore, we treated the water-immersed litter as one group in the subsequent fungal community analyses. Differences in rarefied richness (Chao1) and number of observed OTUs between senescent leaves and submerged litter were tested in the SPSS program by non-parametric Mann–Whitney *U*-test, because the assumption of normal distribution was not met. Boxplots representing median values with interquartile range were drawn. Differences in fungal community composition between senescent leaves and submerged litter were further examined using Permutational Multivariate Analysis of Variance (PERMANOVA; Anderson, 2001). PERMANOVA examines mean differences in community composition among *a priori* defined site groups and partitions dissimilarities across sources of variation in a multivariate data set, yielding tests of significance using a permutation-based *F*-test (Anderson, 2001). The analysis was run based on Bray-Curtis dissimilarities and using 999 permutations. Statistical significances in relative abundances of identified taxon groups between senescent leaves and stream-immersed litter were tested with the Mann–Whitney *U*-test in the SPSS program. We further tested differences in relative abundances of the 54 most commonly occurring fungal taxa between the senescent leaves and submerged litter. Benjamini–Hochberg procedure with *Q* = 0.25 was used to correct *p*-values of multiple tests (Benjamini and Hochberg, 1995). SIMPER (Similarity Percentage Analysis, Clarke, 1993) was used to assess which fungal taxa contributed the most to differences in the community structures. SIMPER analysis was performed with raw abundance data. Differences in ergosterol content between litter samples submerged in acidic or circumneutral streams of the two river basins were studied with *t*-test after examination of normal distribution and equality of variances using Shapiro-Wilk’s and Levene’s tests in R. NMDS, PERMANOVA, and SIMPER were conducted using the R package Vegan (Oksanen et al., 2014).

## Results

Ergosterol analysis, which estimates fungal biomass, indicated that there were 5–13.6 times more fungi present in the stream-immersed litter than in the senescent leaves. The values of ergosterol varied between 64.57 and 176.58 μg/gdw in the litter (Table 2). Significantly more fungal biomass (*t*-test *p* = 0.030) was found in litter submerged in the acidic Oulujoki streams than in the circumneutral Iijoki streams.

**Table 2:** Ergosterol concentrations and relative increase compared to original leaf sample concentration (13.001 μg/gdw) of the litter samples after submergence in streams.

River	River basin^a^	Ergosterol concentration (μg/gdw)^b^	Ergosterol relative increase (fold)
Myl	I	95.19	7.3
Pul	I	64.57	5.0
Lian	I	102.31	7.9
Louh	I	64.91	5.0
Majo	I	87.76	6.8
Toll	I	135.74	10.4
Mus ala	O	150.89	11.6
Mus ylä	O	128.11	9.9
Must	O	132.15	10.2
Pur	O	83.08	6.4
Korp	O	130.57	10.0
Lam	O	176.58	13.6

*Ergosterol content was determined from pooled samples. ^a^I, Iijoki basin; O, Oulujoki basin; ^b^gdw, grams of dry weight.*

**FIGURE 1:**
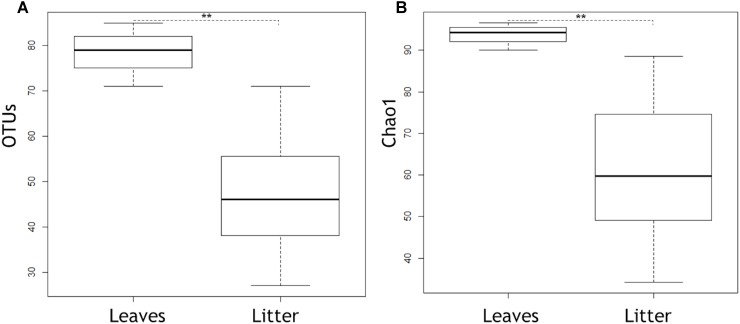
Boxplots showing OTUs **(A)** and Chao1 **(B)** in senescent leaves and submerged litter samples, respectively. Box represents 25–75th percentiles, the line in the middle of the box represents median, and the whiskers represent minimum to maximum values (standard deviation). Asterisks indicate statistically significant differences between senescent leaves and submerged leaf litter in OTUs and Chao1 (*p* = 0.004 for both cases).

**FIGURE 2:**
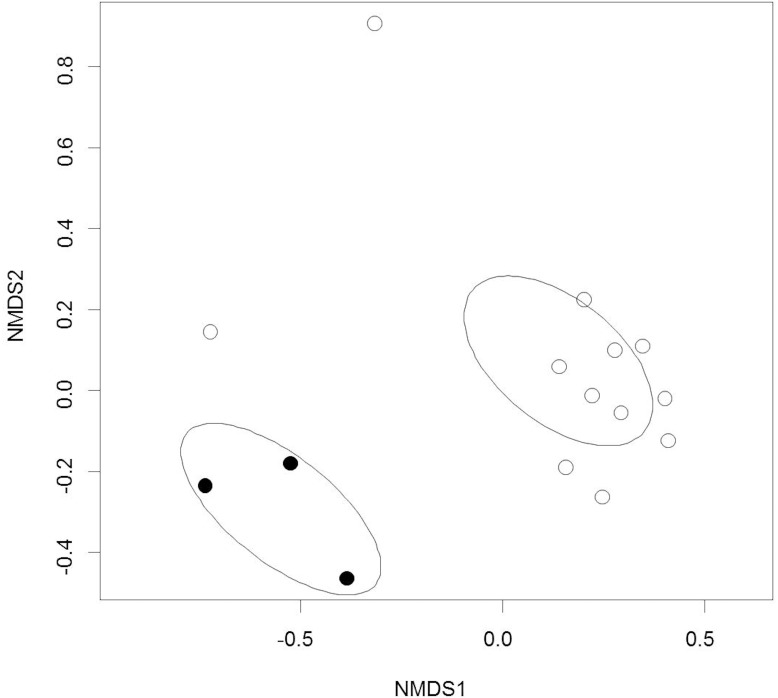
Non-metric Multidimensional Scaling ordination of the fungal communities on alder leaves prior to and after submergence in streams. Black circles = prior to submergence in streams, open circles = after submergence in streams. The ellipses represent the 95% confidence intervals of standard deviations of the group centroids.

Out of the 804 fungal OTUs in the whole dataset, 32 OTUs were classified at species level, and 21 OTUs were classified at genus level. The rest of the OTUs were identified only at family and order levels, which indicates the extent of poorly classified fungi existing in the environments of boreal streams or alder leaves. Overall, the alder leaves harbored a different microbiome prior to than after submergence in the streams (Figure 1, 2). Chao1 values and numbers of observed OTUs were higher to senescent leaves than to submerged litter (Figure 1, Table 3, and Supplementary Tables S2, S3). Samples from senescent leaves and stream-immersed litter were clearly separated in the NMDS ordinations space (Figure 2). PERMANOVA analysis confirmed that the differences between senescent leaves and stream-immersed litter were statistically significant (*F* = 3.843, *p* = 0.02) and explained 22% of variation in composition of fungal communities.

**Table 3:** Statistically significantly different relative abundances of taxa in senescent leaves and stream-immersed litter, as well as the medians of observed OTUs and Chao1, tested by Mann–Whitney *U*-test in SPSS program.

Taxa	Level^a^	Phylum^b^	Senescent leaves^c^	Stream-immersed litter^c^
Leotiomycetes	c	A	0.01^*^	0.087^*^
Xylariales	o	A	0^∗^	0.006^*^
Venturiaceae	f	A	0^∗^	0.014^*^
Mycosphaerellaceae	f	A	0.006^**^	0.001^**^
*Aureobasidium*	g	A	0.131^*^	0.044^*^
*Aspergillus*	g	A	0.291^**^	0^∗∗^
*Cryptococcus*	g	B	0.057^*^	0.017^*^
*Dioszegia*	g	B	0.01^*^	0.001^*^
*Bullera globispora*	s	B	0.007^*^	0.002^*^
*Cryptococcus* sp. FYB_2007a	s	B	0.03^*^	0.009^*^
Median of Observed OTUs and Chao1				
Chao1			93.667^**^	60.749^**^
Observed OTUs^d,e^			78.333^**^	47.417^**^

*^a^c, class, o, order, f, family, g, genus, s, species. ^b^A, Ascomycota, B, Basidiomycota. ^c^Relative abundance. ^∗^p < 0.05, ^∗∗^p < 0.01. ^d^The total number of observed OTUs in the dataset was 804. ^e^The median of OTUs present in each group.*

**FIGURE 3:**
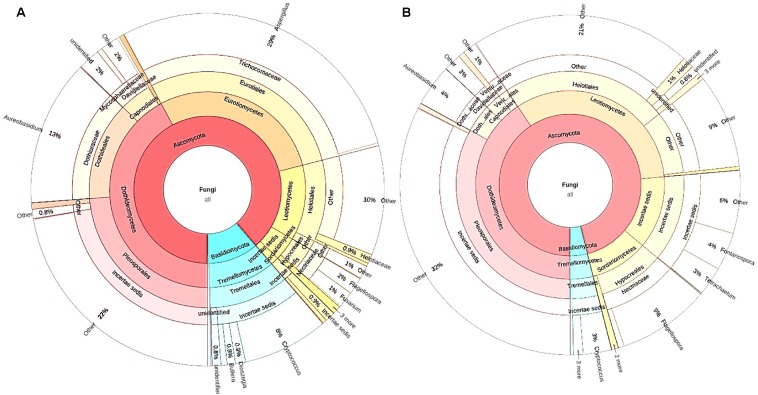
The microbiomes of senescent leaves **(A)** and stream-immersed litter **(B)**. **(A)** The significantly different OTUs in the alder leaves belonged to *Aspergillus*, *Aureobasidium*, and *Cryptococcus*. **(B)** The microbiome of submerged litter consisted significantly more OTUs belonging to Leotiomycetes.

Overall, there were significantly more Basidiomycetes taxa, i.e., *Bullera*, *Dioszegia*, and *Cryptococcus* present in senescent leaves than in submerged litter (Figure 3 and Table 3). At the order/family level, the senescent leaves contained significantly more OTUs belonging to the family Mycosphaerellaceae (0.6%, *p* = 0.005) than litter. Significantly more OTUs belonging to the genera *Aspergillus* (29%, *p* = 0.009), *Aureobasidium* (13%, *p* = 0.02), *Cryptococcus* (8%, *p* = 0.03), and *Dioszegia* (0.9%, *p* = 0.02), were found in the senescent leaves than in stream-immersed litter. At the species level, there were more OTUs identified as *Bullera globispora* (0.7%, *p* = 0.046) and *Cryptococcus* sp. FYB_2007a (3%, *p* = 0.028) in the senescent leaves than in litter (Table 3).

In the submerged litter, we found significantly more OTUs belonging to the class Leotiomycetes (9%, *p* = 0.014), order Xylariales (0.6%, *p* = 0.032), and family Venturiaceae (1.4%, *p* = 0.037) than in senescent leaves (Table 3). The SIMPER analysis supported these results, as *Aspergillus* (29%), *Aureobasidium* (12%), and *Leotiomycetes* (8%) were reported among the taxa responsible for the differing communities in the samples (Supplementary Table S4). Overall, there were no differences observed in the fungal communities of acidic or circumneutral steams (PERMANOVA *F* = 1.257, *p* = 0.18) (Figure 2). When the litter communities of different streams were observed individually, the relative abundance of Basidiomycetes fungi was generally low, with one stream (Korp, Oulujoki basin) being an exception and having a high relative abundance of Basidiomycota, specifically *Cryptococcus* sp. (29%) (Supplementary Figure S1). Within Ascomycota, Dothideomycetes, and Leotiomycetes dominated in the communities and there was high relative abundance of Sordariomycetes (24–40% of *Flagellospora* sp.) in streams such as Mus ylä and Mus ala (Supplementary Figure S1). These two sampling sites belong to the same river system of Oulujoki basin. Furthermore, high relative abundance of *Fontanospora* sp. (13–14%) was found in the litter samples of Toll and Lian streams, which belong to the Iijoki basin, as well as Pur stream, which is part of the Oulujoki basin (Supplementary Figure S1).

**FIGURE 4:**
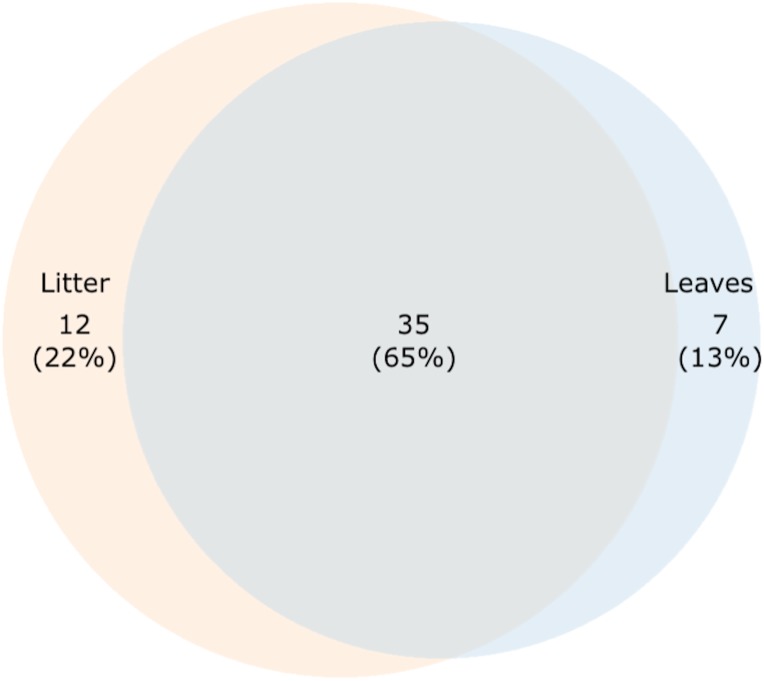
A proportional Venn diagram showing shared and unique OTUs among senescent leaves and stream-immersed litter.

However, to find fungi that might represent both foliar microfungi and aquatic hyphomycetes, we looked for OTUs that were present in both senescent leaves and stream-immersed litter. Shared and unique OTUs between senescent leaves and stream-immersed were plotted on a Venn diagram, where 35 OTUs (65%) were present in all samples (Figure 4). This group comprised OTUs, which were classified based on best BLAST hits (Supplementary Table S5) as *Alatospora* sp. (OTUs nos. 67, 100), *Articulospora* sp. (no. 291), *Caloplaca raesaenenii* (no. 352), *Ciboria shiraiana* (no. 255), *Cora corelleslia* (no. 194), *Cryptococcus* sp. (no. 110), *Daldinia petriniae* (no. 124), *Dioszegia rishiriensis* (no. 82), *Fusarium* sp. (no. 31), *Fusicoccum quercus* (no. 183), *Galactomyces geotrichum* (no. 344), Glomeromycota sp. (nos. 57, 104, 189, 215, 251, 273), Ericoid mycorrhizal sp. (no. 328), *Mycosphaerella* sp. (no. 258), *Phomopsis* sp. (no. 223), *Pyxine endochrysina* (nos. 245, 270), Uncultured endophyte sp. (no. 63), and Uncultured fungus sp. (nos. 59, 89, 172, 195, 238, 242, 314, 321, 322, 336, 339).

Seven OTUs (13%) were found only in senescent leaves (Figure 4 and Supplementary Table S5). These OTUs were classified as Basidiomycota sp. (OTU no. 118), *Dioszegia crocea* (no. 133), Glomeromycota sp. (no. 139), Fungal endophyte sp. (no. 73), *Tremellomycetes* sp. (no. 72), *Wallemia sebi* (no. 108), and Uncultured fungus sp. (no. 167). Twelve OTUs (22%) were identified exclusively in stream-immersed litter (Figure 4 and Supplementary Table S5). These OTUs were classified as *Alatospora* sp. (OTU no. 305), *Amanita wadjukiorum* (no. 43), Basidiomycota sp. (OTU no. 39), *Ciboria carunculoides* (no. 142), *Circinaria esculenta* (no. 152), *Cryptococcus* sp. (no. 15), *Cyphellostereum* sp. (no. 19), *Ditopella aseptatospora* (no. 135), *Kockovaella prillingeri* (nos. 40, 209), *Paramicrothyrium* sp. (no. 123), and Uncultured fungus sp. (no. 28).

## Discussion

Fungal biomass on litter is often reported to decline in anthropogenically acidified streams (Ely et al., 2010; Valett and Ely, 2019). Contrary to expectations, there was significantly more fungal biomass in litter submerged in the pristine acidic streams and the fungal communities between streams were similar. Alder leaves generally comprised different fungal communities prior to immersion in stream water, being richer by Chao1 values and containing significantly more Basidiomycetes taxa, i.e., *Bullera, Dioszegia*, and *Cryptococcus*. The litter had significantly more OTUs belonging to the class Leotiomycetes. However, our main attention was drawn to the group of fungal OTUs present both in the senescent leaves and the litter.

A few species of foliar microfungi have previously been identified as leaf decomposers in the terrestrial environment (Promputtha et al., 2007; Sun et al., 2011; Unterseher et al., 2013). Even if not directly participating in the decomposition process, they can significantly affect the decomposition rates (Omacini et al., 2004; Lemons et al., 2005; Osono and Hirose, 2009; Purahong and Hyde, 2011). Although our study was limited to only three replicates for senescent leaves, we demonstrate that foliar microfungi of terrestrial origin colonize alder litter more than anticipated in the aquatic environment. We found a group of 35 OTUs (65%) representing foliar microfungi and aquatic transient fungi. This group comprised some OTUs belonging to species or genera that were abundant in the alder leaves prior to submergence in water. These OTUs were *Cryptococcus* sp. (no. 110), *Dioszegia rishiriensis* (no. 82), and *Mycosphaerella* sp. (no. 258). *Cryptococcus* and *Dioszegia* are closely related species that have been detected in a number of environments, including plant leaves, soil, water (Renker et al., 2004), and litter (Wuczkowski et al., 2005). Sampaio et al. (2007) have studied the presence of yeasts in the litter of several tree species in streams, and found two species of *Cryptococcus*, *C. laurentii* and *C. albidus*, present in the litter of alder. Although *D. rishiriensis* has originally been isolated from soil (Takashima et al., 2011), many members of *Dioszegia* spp. are reported as endo/epiphytes (Jumpponen and Jones, 2009), decomposers in forest litter (Wuczkowski et al., 2005), and have been detected in aquatic cold environments (De García et al., 2007).

The *Mycosphaerella* genus includes endophytic (Moreno et al., 2011) and plant parasitic-pathogenic (Carnegie et al., 1997) strains. Unterseher et al. (2013) have identified a *Mycosphaerella* sp. both in the leaves and litter of beech, but we found no earlier reports of *Mycosphaerella* as an aquatic fungus or hyphomycetes. Similarly, the species *Daldinia petriniae* (no. 124) has earlier been isolated from litter of deciduous forest, although without ligninolytic activity (Cha et al., 2011), and some members of *Daldinia* have been identified as endophytes (Chen et al., 2011), but no water-associated reports have been found. Other taxa found in the submerged litter and having not been previously identified as aquatic fungi include *Phomopsis*, *Ciboria shiraiana*, and *Fusicoccum quercus*. *Phomopsis* spp. (no. 223) are widely identified as endophytes in leaves of trees (Murali et al., 2006), such as alder (Sieber et al., 1991), and are documented to have the capacity for leaf degradation (Chen et al., 2010), but *Phomopsis* spp. can also cause severe infections as plant pathogens (Pscheidt and Pearson, 1989). *Ciboria shiraiana* (no. 255) is a pathogen on mulberry (Hong et al., 2007) and *Fusicoccum quercus* (no. 183) on oak (Kowalski, 1991). *F. quercus* has also been reported as an oak endophyte (Ragazzi et al., 2003).

Several OTUs, identified in both leaves and litter, were classified within the phylum Glomeromycota (nos. 57, 104, 189, 215, 251, 273), which is known to host members forming the arbuscular mycorrhiza (Schüßler et al., 2001) even in submerged conditions (Clayton and Bagyaraj, 1984). This result agrees with the earlier studies that suggested a connection between aquatic hyphomycetes and mycorrhizal roots (Murat et al., 2005; Abadie et al., 2006; Tedersoo et al., 2007; Stark et al., 2009; Elena et al., 2010). Glomeromycota were recently identified in the litter of oak at the later successional stages (Vořšková and Baldrian, 2013). Soil-borne asexual spores of Glomeromycota are normally dispersed by the wind after drought periods and are found in soil dust deposits (Warner et al., 1987). However, what makes our finding intriguing is that the Glomeromycota are considered obligate symbionts in plant roots (Schüßler and Walker, 2010), and they are not generally found in leaves. We found only one paper reporting Glomeromycota as a foliar endophyte in a plant species of Antarctica (Rosa et al., 2010). Another interesting group of OTUs found in all samples were classified as lichenicolous fungi, *Caloplaca raesaenenii* (no. 352) (Vondrak et al., 2012), the newly identified *Cora corelleslia* (no. 194) (Vargas et al., 2014), as well as the well-known *Pyxine endochrysina* (no. 245) (Yosioka et al., 1972). Although no reports are found on these species as aquatic hyphomycetes, saprotrophs, or foliar microfungi, other lichenicolous fungi are found in plant leaves as endophytes (U’Ren et al., 2010).

The rest of the OTUs found in all samples belonged to species or genera that are more or less typical for water environments. *Fusarium* species (no. 31) are cosmopolitans, being identified typically as plant pathogens (Scher and Baker, 1982), endophytes (Kajula et al., 2010), and in alder litter submerged in water (Ingold, 1942). *Galactomyces geotrichum* (no. 344) is found as an endophyte in the roots of the water plant *Trapa japonica* (Waqas et al., 2014) and as an aquatic fungus in the sediments of mangrove soil (Arfi et al., 2012). *Alatospora* sp. (OTUs no. 67, 100) and *Articulospora* sp. (no. 291) have earlier been reported as both aquatic hyphomycetes and root endophytes (Sridhar and Bärlocher, 1992; Sati and Belwal, 2005).

Operational taxonomic units found solely in the stream litter represent aquatic resident fungi. What was striking within this group was the presence of Basidiomycota, namely *Cryptococcus* sp. (no. 15), *Amanita wadjukiorum* (no. 43), *Kockovaella prillingeri* (no. 40, 209), and *Basidiomycota* sp. (OTU no. 39). Basidiomycetes are rare in the aquatic environment, in general (Shearer et al., 2007), but earlier studies have shown that Basidiomycetes sporadically occur in the freshwater environment as asexually reproducing anamorphs (Jones and Choecyklin, 2007). *K. prillingeri* is a species recently identified from bromeliad tank water (Gomes et al., 2016). *A. wadjukiorum* is a mycorrhizal macrofungus (Davison et al., 2013) and spores of *A. muscaria* have been found in water (Czeczuga and Muszyńska, 2004). Another uncommon aquatic fungus was *Ciboria carunculoides* (no. 142), which has previously been reported as a pathogen in mulberry (Hong et al., 2007). *Paramicrothyrium* sp. (no. 123) and *Ditopella aseptatospora* (no. 135) have previously been reported as plant-associated microfungi (Wu et al., 2011; Tian et al., 2018). *Circinaria esculenta* (no. 152) and *Cyphellostereum* sp. (no. 19) are lichenicolous fungi with no previous references to water (Høiland, 1976; Sohrabi et al., 2013). The only typical aquatic hyphomycetes (Sati and Belwal, 2005) in this group was the OTU classified as *Alatospora* (OTU no. 305).

Sun et al. (2011) identified, depending on method, 35 or 9 out of 58 endophytic taxa being capable of leaf decomposition. In general, the proportion of foliar microfungi present in decomposer fungal communities in terrestrial conditions has been estimated to be 2–100% (Osono, 2006). In our study on aquatic environment, 65% of fungi originated from the tree host. This reflects the extent to which diversity of foliar microfungi can contribute to fungal diversity in submerged litter. Therefore, forest management activities not only reduce diversity of foliar microfungi (Ganley and Newcombe, 2006), but also fungal diversity in streams. Senescent leaves intimately link the two ecosystems, terrestrial and aquatic, by transporting fungi from vegetation to the water, where they colonize various niches as biofilms (Podolich et al., 2009; Dang and Lovell, 2016). However, while DNA sequencing allows detection and identification of microbial species, the method produces no functional information, i.e., which species are active decomposers in the litter fungal community. For example, sequencing of RNA or stable isotope probing of communities responsible of alder leaf decomposition would provide evidence on the functional roles of different fungal members. In the future, these methods will elucidate the extent to which foliar microfungi contribute to litter decomposition in stream ecosystems.

## Conclusion

Our study demonstrates that the fungal community of water-submerged litter contains foliar microfungi originating from terrestrial environment. The community of water-submerged litter had 35 OTUs (65%) in common with senescent leaves. This group consisted of taxa typically identified as foliar microfungi, such as *Cryptococcus*, *Dioszegia*, and *Mycosphaerella*, taxa known as aquatic hyphomycetes, such as *Fusarium*, *Alatospora*, and *Articulospora*, but also taxa uncommon to plant leaves, such as Glomeromycota, and taxa uncommon to aquatic environment, such as lichenicolous fungi. However, further studies are needed on the functional roles of each fungal species in the leaf decomposition process in aquatic conditions.

## Author Contributions

PK analyzed the sequence data in QIIME. MVT supervised PK and analyzed the sequence data. MT processed the samples and raw data sequences, and together with HM performed statistical analyses. AM performed ergosterol analysis, modified and commented the manuscript. HM planned the study, supervised MT, and modified and commented the manuscript. AMP analyzed data with others and wrote the manuscript.

## Conflict of Interest Statement

The authors declare that the research was conducted in the absence of any commercial or financial relationships that could be construed as a potential conflict of interest.

## Supplementary Material

FIGURE S1 A bar chart representing fungal microbiomes of alder litter submerged in 12 different streams, Myl, Pul, Lian, Louh, Majo, and Toll, of the Iijoki basin, and Mus ala, Mus ylä, Must, Pur, Korp, and Lam of the Oulujoki basin.

TABLE S1 Habitat and water chemistry characteristics of the study sites. Substrate size was based on ten estimates of the % cover of ten substrate classes: (0) organic matter, (1) sand (diameter 0.25–2 mm), (2) fine gravel (2–6 mm), (3) coarse gravel (6–16 mm), (4) small pebble (16–32 mm), (5) large pebble (32–64 mm), (6) small cobble (64–128 mm), (7) large cobble (128–256 mm), (8) boulder (256–400 mm), (9) large boulder and bed rock (>400 mm).

TABLE S2 Alpha diversity indices (Chao1, Observed OTUs) of the fungal communities in alder leaf and stream-immersed litter samples.

TABLE S3 Statistical significances of the Observed OTUs and Chao1 richness of the fungal communities in senescent leaf and submerged litter samples, tested by the Mann–Whitney *U*-test in SPSS.

TABLE S4 Taxa responsible for the differing communities in the samples tested by the SIMPER analysis.

TABLE S5 Operational taxonomic units found in the various environments; senescent leaves, litter, or both, as presented in the Venn diagram (Figure 4), and corresponding closest BLAST hits.
