# Supplementary figures and images for: Fungi Originating From Tree Leaves Contribute to Fungal Diversity of Litter in Streams
Source: Front Microbiol. 2019 Apr 2;10:651. doi: 10.3389/fmicb.2019.00651 (PMC6454979; doi:10.3389/fmicb.2019.00651)

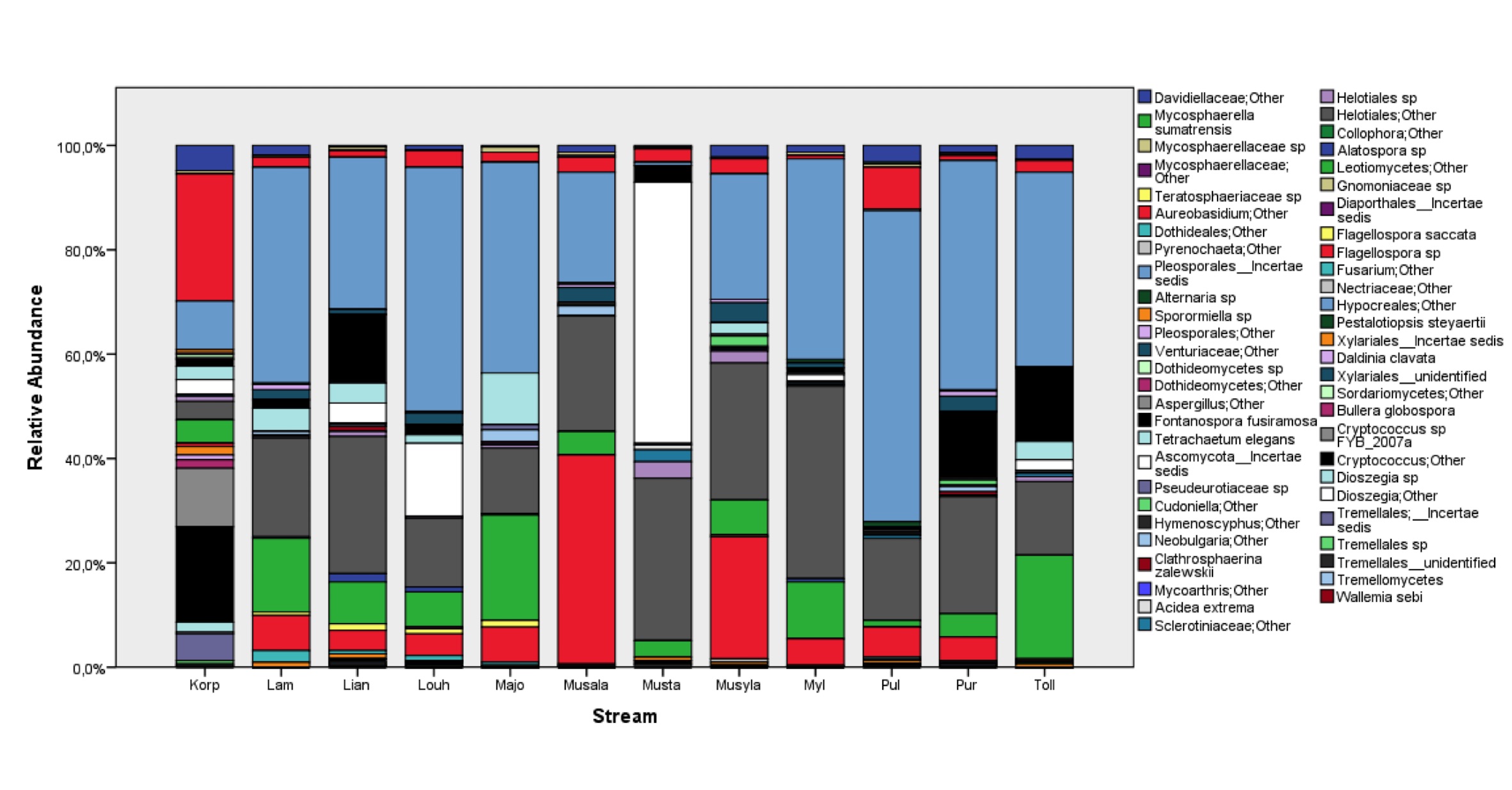

Supplement: FIGURE S1 — A bar chart representing fungal microbiomes of alder litter submerged in 12 different streams, Myl, Pul, Lian, Louh, Majo, and Toll, of the Iijoki basin, and Mus ala, Mus ylä, Must, Pur, Korp, and Lam of the Oulujoki basin. [file Image_1.JPEG]
