# Supplementary material for: Fungi Originating From Tree Leaves Contribute to Fungal Diversity of Litter in Streams
Source: Front Microbiol. 2019 Apr 2;10:651. doi: 10.3389/fmicb.2019.00651 (PMC6454979; doi:10.3389/fmicb.2019.00651)
Supplement: TABLE S1 — Habitat and water chemistry characteristics of the study sites. Substrate size was based on ten estimates of the % cover of ten substrate classes: (0) organic matter, (1) sand (diameter 0.25–2 mm), (2) fine gravel (2–6 mm), (3) coarse gravel (6–16 mm), (4) small pebble (16–32 mm), (5) large pebble (32–64 mm), (6) small cobble (64–128 mm), (7) large cobble (128–256 mm), (8) boulder (256–400 mm), (9) large boulder and bed rock (>400 mm). [file Table_1.DOCX]

Table S1. Habitat and water chemistry characteristics of the study sites. Substrate size was based on ten estimates of the % cover of ten substrate classes: (0) organic matter, (1) sand (diameter 0.25 mm- 2 mm), (2) fine gravel (2 mm – 6 mm), (3) coarse gravel (6 mm – 16 mm), (4) small pebble (16 mm – 32 mm), (5) large pebble (32 mm – 64 mm), (6) small cobble (64 mm – 128 mm), (7) large cobble ( 128 mm – 256 mm), (8) boulder (256 mm – 400 mm), (9) large boulder and bed rock (> 400 mm).

| **Stream** | Area^a^ | Velocity  (m/s) | **Depth**  (cm**)** | **Width**  (m) | **Moss cover** (%) | **Shading**  (%) | **Particle size** | **tot-P**  (µg/L) | **pH** | **Fe**  (µg/L) | **Al**  (µg/L) | **Cu**  (µg/L) | **Zn**  (µg/L) | **DOC**  (mg/L) | **Alkalinity**  (mmol/L) | **Conductivity**  (mS/m) |
| --- | --- | --- | --- | --- | --- | --- | --- | --- | --- | --- | --- | --- | --- | --- | --- | --- |
| Myl | I | 0.23 | 41 | 5.0 | 87 | 20 | 6.8 | 9 | 6.7 | 170 | 48 | 0.13 | 1.56 | 5.2 | 0.18 | 2.6 |
| Pul | I | 0.22 | 26 | 1.5 | 71 | 39 | 7.2 | 7 | 6.6 | 620 | 76 | 0.1 | 2.11 | 12 | 0.20 | 3.0 |
| Lian | I | 0.21 | 23 | 4 | 84 | 48 | 5.8 | 10 | 6.3 | 590 | 120 | 0.17 | 2.12 | 17 | 0.14 | 2.5 |
| Louh | I | 0.13 | 25 | 1.5 | 67 | 41 | 7.8 | 15 | 6.5 | 960 | 149 | 0.16 | 2.71 | 18 | 0.13 | 2.5 |
| Majo | I | 0.19 | 18 | 1.5 | 78 | 48 | 4.7 | 14 | 6.8 | 1000 | 93 | 0.12 | 2.49 | 11 | 0.18 | 3.6 |
| Toll | I | 0.12 | 18 | 0.5 | 65 | 43 | 5.9 | 12 | 5.9 | 830 | 74 | 0.12 | 3.07 | 20 | 0.08 | 1.9 |
| Mus ala | O | 0.47 | 26 | 8.9 | 73 | 1 | 7.6 | 22 | 6.3 | 1600 | 298 | 1.00 | 15.7 | 10 | 0.05 | 3.2 |
| Mus ylä | O | 0.46 | 25 | 8.7 | 78 | 0 | 7.3 | 24 | 5.5 | 1800 | 305 | 0.96 | 18.6 | 10 | 0.02 | 3.2 |
| Must | O | 0.34 | 24 | 0.7 | 18 | 48 | 5.2 | 18 | 4.2 | 1700 | 331 | 0.38 | 10.3 | 40 | -0.02 | 4.6 |
| Pur | O | 0.19 | 19 | 0.9 | 55 | 5 | 6.2 | 12 | 5.8 | 840 | 229 | 0.67 | 7.69 | 16 | 0.04 | 3.2 |
| Korp | O | 0.28 | 22 | 0.7 | 55 | 25 | 7.3 | 17 | 4.8 | 2200 | 305 | 0.65 | 15.6 | 25 | -0.02 | 2.6 |
| Lam | O | 0.47 | 31 | 5.0 | 46 | 42 | 7.3 | 24 | 5.4 | 1800 | 304 | 0.99 | 17.3 | 11 | 0.02 | 3.2 |

^a^I= Iijoki basin, O=Oulujoki basin
