# Supplementary material for: Fungi Originating From Tree Leaves Contribute to Fungal Diversity of Litter in Streams
Source: Front Microbiol. 2019 Apr 2;10:651. doi: 10.3389/fmicb.2019.00651 (PMC6454979; doi:10.3389/fmicb.2019.00651)
Supplement: TABLE S2 — Alpha diversity indices (Chao1, Observed OTUs) of the fungal communities in alder leaf and stream-immersed litter samples. [file Table_2.DOCX]

| Sample | Chao1 | Observed OTUs |
| --- | --- | --- |
| leaf_A | 96.7 | 85.0 |
| leaf_B | 94.3 | 71.0 |
| leaf_C | 90.0 | 79.0 |
| KorpT | 88.5 | 71.0 |
| LianT | 78.1 | 63.0 |
| MylT | 71.2 | 56.0 |
| PurT | 59.5 | 55.0 |
| LouhT | 79.3 | 53.0 |
| TollT | 60.1 | 47.0 |
| LamT | 63.2 | 45.0 |
| MajoT | 57.2 | 42.0 |
| PulT | 50.1 | 39.0 |
| MusylaT | 48.0 | 37.0 |
| MustaT | 39.6 | 34.0 |
| MusalaT | 34.2 | 27.0 |

Table S2. Alpha diversity indices (Chao1, Observed OTUs) of the fungal communities in senescent leaf and stream-immersed litter samples.
