# Supplementary material for: Fungi Originating From Tree Leaves Contribute to Fungal Diversity of Litter in Streams
Source: Front Microbiol. 2019 Apr 2;10:651. doi: 10.3389/fmicb.2019.00651 (PMC6454979; doi:10.3389/fmicb.2019.00651)
Supplement: TABLE S3 — Statistical significances of the Observed OTUs and Chao1 richness of the fungal communities in senescent leaf and submerged litter samples, tested by the Mann–Whitney U-test in SPSS. [file Table_3.docx]

Table S3. Statistical significances of the Observed OTUs and Chao1 richness of the fungal communities in senescent leaf and submerged litter samples, tested by the Mann-Whitney U test in SPSS.

| **Sample** | **Observed OTUs** | | **Chao1** | |
| --- | --- | --- | --- | --- |
|  | **Mann-Whitney U** | ***p*-value** | **Mann-Whitney U** | ***p*-value** |
| Leaves × Litter | 0.500 | 0.004 | 0.00 | 0.004 |
