# Supplementary material for: Fungi Originating From Tree Leaves Contribute to Fungal Diversity of Litter in Streams
Source: Front Microbiol. 2019 Apr 2;10:651. doi: 10.3389/fmicb.2019.00651 (PMC6454979; doi:10.3389/fmicb.2019.00651)
Supplement: TABLE S4 — Taxa responsible for the differing communities in the samples tested by the SIMPER analysis. [file Table_4.DOCX]

| **Taxa** | **Contribution (%)** |
| --- | --- |
| Leotiomycetes | 7.8 |
| Pleosporales | 13.5 |
| *Aspergillus* | 29.7 |
| *Aureobasidium* | 11.8 |
| *Cryptococcus* | 3.0 |
| *Fusarium* | 1.3 |
| *Cryptococcus* sp FYB_2007a | 2.6 |
| *Fontanospora fusiramosa* | 1.4 |

Table S4. Taxa responsible for the differing communities in the samples tested by the SIMPER analysis.
