# Supplementary material for: Fungi Originating From Tree Leaves Contribute to Fungal Diversity of Litter in Streams
Source: Front Microbiol. 2019 Apr 2;10:651. doi: 10.3389/fmicb.2019.00651 (PMC6454979; doi:10.3389/fmicb.2019.00651)
Supplement: TABLE S5 — Operational taxonomic units found in the various environments; senescent leaves, litter, or both, as presented in the Venn diagram (Figure 4), and corresponding closest BLAST hits. [file Table_5.DOCX]

Table S5. OTUs found in the various environments; senescent leaves, litter, or both, as presented in the Venn diagram (Figure 4), and corresponding closest BLAST hits.

| **OTU No.** | **Classification** | **Max. Score** | **Total Score** | **Query coverage (%)** | **E-value** | **Max. Identity (%)** | **Accession** |
| --- | --- | --- | --- | --- | --- | --- | --- |
| **Leaves** | | | | | | | |
| 72 | *Tremellomycetes* sp. | 220 | 308 | 74% | 1e-53 | 93% | KT728331.1 |
| 73 | Fungal endophyte | 233 | 233 | 62% | 2e-57 | 92% | KR016399.1 |
| 108 | *Wallemia sebi* | 226 | 226 | 56% | 3e-55 | 94% | MF098689.1 |
| 118 | *Basidiomycota* sp. | 228 | 314 | 75% | 7e-56 | 94% | KT160981.1 |
| 133 | *Dioszegia crocea* | 219 | 305 | 76% | 4e-53 | 92% | AJ581080.1 |
| 139 | Glomeromycota sp. | 230 | 230 | 85% | 2e-56 | 85% | JN685239.1 |
| 167 | Uncultured fungus | 224 | 312 | 74% | 9e-55 | 93% | KF800549.1 |
| **Leaves and Litter** | | | | | | | |
| 31 | *Fusarium* sp. | 226 | 226 | 56% | 3e-55 | 94% | KX343029.1 |
| 57 | *Glomeromycota* sp. | 230 | 230 | 85% | 2e-56 | 85% | JN685239.1 |
| 59 | Uncultured fungus | 224 | 310 | 72% | 9e-55 | 94% | JX336429.1 |
| 63 | Uncultured endophyte | 231 | 231 | 57% | 6e-57 | 94% | FM200696.1 |
| 67 | *Alatospora* sp. | 224 | 312 | 72% | 9e-55 | 94% | KT728296.1 |
| 82 | *Dioszegia rishiriensis* | 222 | 316 | 75% | 3e-54 | 93% | KY103358.1 |
| 89 | Uncultured fungus | 226 | 314 | 78% | 3e-55 | 92% | KF800591.1 |
| 100 | *Alatospora* sp. | 224 | 312 | 72% | 9e-55 | 94% | KT728296.1 |
| 104 | Glomeromycota sp. | 230 | 230 | 85% | 2e-56 | 85% | JN685239.1 |
| 110 | *Cryptococcus* sp. | 230 | 230 | 58% | 2e-56 | 93% | JF449764.1 |
| 124 | *Daldinia petriniae* | 220 | 220 | 54% | 1e-53 | 94% | KX589226.1 |
| 172 | Uncultured fungus | 228 | 228 | 56% | 7e-56 | 94% | KU582089.1 |
| 183 | *Fusicoccum quercus* | 220 | 308 | 75% | 1e-53 | 93% | KY367512.1 |
| 189 | Glomeromycota sp*.* | 233 | 233 | 85% | 2e-57 | 86% | JN685237.1 |
| 194 | *Cora corelleslia* | 224 | 314 | 77% | 9e-55 | 93% | KX772433.1 |
| 195 | Uncultured fungus | 233 | 233 | 56% | 2e-57 | 95% | HM770096.1 |
| 215 | Glomeromycota sp*.* | 224 | 224 | 85% | 9e-55 | 85% | JN685239.1 |
| 223 | *Phomopsis* sp. | 224 | 224 | 54% | 9e-55 | 94% | LC150821.1 |
| 238 | Uncultured fungus | 228 | 228 | 56% | 7e-56 | 94% | KU582089.1 |
| 242 | Uncultured fungus | 228 | 312 | 74% | 6e-56 | 94% | JX349777.1 |
| 245 | *Pyxine endochrysina* | 224 | 224 | 58% | 7e-55 | 92% | AY498680.1 |
| 251 | Glomeromycota sp. | 232 | 232 | 85% | 5e-57 | 83% | JN685237.1 |
| 255 | *Ciboria shiraiana* | 239 | 325 | 73% | 3e-59 | 95% | HQ833461.1 |
| 258 | *Mycosphaerella* sp. | 226 | 303 | 72% | 2e-55 | 94% | DQ984133.1 |
| 270 | Uncultured fungus | 222 | 306 | 78% | 3e-54 | 93% | KU582196.1 |
| 273 | Glomeromycota sp. | 228 | 228 | 85% | 7e-56 | 85% | JN685237.1 |
| 291 | *Articulospora* sp. | 220 | 316 | 74% | 1e-53 | 94% | KP234352.1 |
| 314 | Uncultured fungus | 226 | 312 | 77% | 3e-55 | 93% | JX365178.1 |
| 321 | Uncultured fungus | 226 | 314 | 78% | 3e-55 | 92% | KF800591.1 |
| 322 | Uncultured fungus | 226 | 312 | 77% | 3e-55 | 93% | JX365178.1 |
| 328 | Ericoid mycorrhizal sp. | 224 | 224 | 55% | 9e-55 | 94% | AY599242.1 |
| 336 | Uncultured fungus | 224 | 312 | 72% | 9e-55 | 94% | JX346561.1 |
| 339 | Uncultured fungus | 226 | 312 | 78% | 3e-55 | 92% | JX327625.1 |
| 344 | *Galactomyces geotrichum* | 268 | 268 | 65% | 4e-68 | 94% | KF225048.1 |
| 352 | *Caloplaca raesaenenii* | 226 | 312 | 75% | 3e-55 | 93% | HM582183.1 |
| **Litter** | | | | | | | |
| 15 | *Cryptococcus* sp. | 230 | 230 | 58% | 2e-56 | 93% | JF449764.1 |
| 19 | *Cyphellostereum* sp. | 222 | 310 | 77% | 3e-54 | 93% | KY861615.1 |
| 28 | Uncultured fungus | 226 | 310 | 74% | 3e-55 | 93% | KU164037.1 |
| 39 | *Basidiomycota* sp. | 226 | 323 | 77% | 3e-55 | 93% | KP714641.1 |
| 40 | *Kockovaella prillingeri* | 224 | 305 | 74% | 9e-55 | 93% | KY103856.1 |
| 43 | *Amanita wadjukiorum* | 222 | 306 | 82% | 3e-54 | 92% | NR_137116.1 |
| 123 | *Paramicrothyrium* sp. | 224 | 224 | 56% | 9e-55 | 93% | KU747771.1 |
| 135 | *Ditopella aseptatospora* | 220 | 220 | 56% | 1e-53 | 93% | KY883769.1 |
| 142 | *Ciboria carunculoides* | 239 | 325 | 74% | 3e-59 | 95% | HQ833452.1 |
| 152 | *Circinaria esculenta* | 228 | 228 | 57% | 7e-56 | 93% | JQ797510.1 |
| 209 | *Kockovaella prillingeri* | 224 | 305 | 74% | 9e-55 | 93% | KY103856.1 |
| 305 | Uncultured *Alatospora* sp. | 224 | 312 | 72% | 9e-55 | 94% | KT728296.1 |
